# Association of Thyroid-Stimulating Hormone and Thyroid Hormones with Cardiometabolic Risk Factors in Euthyroid Children and Adolescents Aged 10–18 Years: A Population-Based Study

**DOI:** 10.1038/s41598-019-51963-7

**Published:** 2019-10-29

**Authors:** Cheol Gyu Ma, Young Suk Shim

**Affiliations:** Department of Pediatrics, Hallym University Dongtan Sacred Heart Hospital, Hwaseong, Korea

**Keywords:** Thyroid diseases, Risk factors

## Abstract

Recent evidence indicates that low-normal thyroid function test results within the reference ranges may be related to increased cardiometabolic risk factors. The current study aimed to evaluate the relationship between thyroid function using thyroid-stimulating hormone (TSH) and free thyroxine (FT4) and cardiometabolic risk factors and the clustering of these risk factors (metabolic syndrome) in euthyroid children and adolescents. A total of 250 euthyroid children and adolescents aged 10–18 years were included using data from the Korea National Health and Nutrition Examination Survey (KNHANES) 2015. In the unadjusted correlation analyses, TSH was positively correlated with glucose (r = 0.172, *P* = 0.006), hemoglobin A1c (HbA1c) (r = 0.149, *P* = 0.018), insulin (r = 0.144, *P* = 0.023), homeostatic model assessment for insulin resistance (HOMA-IR) (r = 0.163, *P* = 0.010), and triglyceride (TG) (r = 0.155, *P* = 0.014), whereas FT4 was negatively associated with the waist circumference (WC) standard deviation score (SDS) (r = −0.134, *P* = 0.035), body mass index (BMI) SDS (r = −0.126, *P* = 0.046), insulin (r = −0.219, *P* < 0.001), and HOMA-IR (r = −0.211, *P* < 0.001). In the multiple linear regression analysis, TSH was positively associated with glucose (β = 1.179, *P* = 0.021), HbA1c (β = 0.044, *P* = 0.039), and TG (β = 8.158, *P* = 0.041) after adjustment for possible confounders. FT4 was negatively associated with serum fasting insulin (β = −5.884, *P* = 0.017) and HOMA-IR (β = −1.364, *P* = 0.023) in the multiple linear regression analysis. Boys and girls with elevated glucose levels had a higher adjusted mean TSH level compared to those without elevated glucose levels after controlling for confounding factors in the analysis of covariance (2.16 mIU/L vs 3.88 mIU/L, *P* = 0.004). Our results suggest that higher TSH and/or lower FT4 levels, even within the reference ranges, may be related to increased cardiometabolic risk factors.

## Introduction

Cardiovascular disease, which caused approximately 17.6 million deaths in 2016, is an important cause of morbidity and mortality worldwide^1^. Although the disease is considered a major concern in adulthood, the age of onset has tended to be younger in recent decades^2^. Both risk factors related to cardiovascular disease, including abdominal obesity, increased blood pressure (BP), abnormal glucose regulation, decreased insulin sensitivity, dyslipidemia, and clustering of these risk factors, which is called metabolic syndrome (MetS), may begin early in life^3^. Atherosclerosis can also develop in childhood^4^. Cardiometabolic risk factors in childhood and adolescence may be related to cardiovascular disease and type 2 diabetes mellitus (T2DM) in adulthood^5^. Early identification of and intervention in modifiable cardiometabolic risk factors during these early periods is valuable for preventing the development of future disease.

Thyroid hormone and its regulating hormone (thyroid-stimulating hormone, TSH) are not only required for regulating metabolic processes essential for normal growth and development but also are essential for controlling metabolism^6^. Abnormal changes in these hormones can affect adverse health conditions. The relationship between overt hypothyroidism and cardiometabolic diseases and its morbidity and mortality have been well established in many studies^7,8^. Based on the previous studies, the prevalence of overt hypothyroidism is estimated to range from 0.4% to 3.7% in the general population^9–11^. Subclinical hypothyroidism (SCH) is defined as elevated serum TSH levels exceeding the limit of the reference range for age and sex, while the concentrations of serum free thyroxine (FT4) and free triiodothyroxine (FT3) remain within the reference ranges; SCH is more common than overt hypothyroidism, and the prevalence ranges from 4.6% to 9.0% in the adult population^9,12,13^ and from 4.6% to 13.9% in children and adolescents^14,15^. SCH is considered to be related to a variety of health concerns, including cardiometabolic diseases in recent reports^16,17^. In addition, emerging evidence indicates that higher TSH and/or lower free thyroid hormone levels within the reference ranges may be related to increased cardiometabolic risk in recent studies^18–20^.

The present study was conducted to investigate the relationships of thyroid hormone and TSH with cardiometabolic risk factors, including BP, glucose, insulin, insulin resistance indices, and cholesterol profiles, in euthyroid boys and girls aged 10–18 years using nationally representative Korean data. In addition, we evaluated the differences in levels of TSH and FT4 according to the presence of MetS and its components.

## Materials and Methods

### Subjects

We analyzed data from the Korea National Health and Nutrition Examination Survey (KNHANES) 2015. The KNHANES is conducted to investigate the health and nutrition status of Koreans using a multistage clustered probability sampling method. This national survey has a cross-sectional design and consists of three parts, namely, a health interview, health examination, and nutritional survey. The survey is implemented under the initiative of the Korea Centers for Disease Control and Prevention (KCDC)^21^. Further details regarding the KNHANES have been presented previously^22^. The dataset is openly available to the public and is accessible through the internet (http://knhanes.cdc.go.kr). All voluntary subjects in this survey signed informed consent forms.

Among the 7,380 participants in the KNHANES 2015, we enrolled 748 boys and girls aged 10–18 years in the preliminary analysis. Of these, we excluded subjects who had not completed physical and laboratory examinations (*n* = 427). Children and adolescents who exhibited TSH levels <0.4 mIU/L or >4.0 mIU/L were excluded (*n* = 70). Subjects who had FT4 levels <0.9 ng/mL or >2.0 ng/mL were excluded (*n* = 1). We also excluded children and adolescents diagnosed with T2DM or those currently using medication for T2DM or thyroid disease (*n* = 0). Ultimately, we analyzed 250 participants (131 boys and 119 girls) in the present study. The Institutional Review Boards of the KCDC approved the study protocols of the KNHANES 2015. All subjects and parents and/or legal guardians (age of participants <18 years) provided informed consent. All methods in KNHANES were performed in accordance with relevant guidelines and regulations.

### Measurements

The height (cm) and weight (kg) were determined using an electronic stadiometer (SECA, Germany) and an electronic scale (G-TECH, Korea). The body mass index (BMI, kg/m^2^) was calculated as the weight in kilograms divided by the square of the height in meters. The waist circumference (WC, cm) was determined using a calibrated tape (SECA, Germany) at the median line between the lower border of the rib cage and the upper border of the iliac crest. For the analyses, the height, weight, BMI and WC were converted into standard deviation scores (SDSs) using LMS methods (measured value/M)^1/L^/LS) according to the 2007 Korean ref.^23^. The systolic and diastolic blood pressure (SBP and DBP, mmHg) were measured using a standard mercury sphygmomanometer (Baum, USA) with an appropriately sized cuff. The assessment was performed three times at two minute intervals. For the analyses, the mean of the second and third measurements was used for the SBP and DBP.

Blood samples were collected year-round after at least 8 hours of fasting. The analyses were performed at the central laboratory (NeoDin Medical Institute, Korea) within 24 hours of blood sampling. The levels of glucose, total cholesterol (T-C), high-density lipoprotein cholesterol (HDL-C), and triglycerides (TG) were measured using an automatic analyzer (Hitachi 7600; Hitachi, Japan). The low-density lipoprotein cholesterol (LDL-C) level was calculated using Friedewald’s equation (LDL-C = T-C − HDL-C − TG/5)^24^. Non-HDL-C was determined as T-C minus HDL-C. The TG to HDL-C ratio (TG/HDL ratio) was assessed as TG divided by HDL-C. Hemoglobin A1c (HbA1c, %) levels were determined using liquid chromatography (HLC-723G7; Tosho, Japan). Insulin levels were assessed using an immunoradiometric assay (1470 Wizard Gamma Counter; Perkin-Elmer, Finland). The homeostatic model assessment for insulin resistance (HOMA-IR) was used as an index of insulin resistance and was assessed according to the following equation^25^:

$${\rm{HOMA}} \mbox{-} {\rm{IR}}=[{\rm{fasting}}\,{\rm{insulin}}\,({\rm{\mu }}{\rm{U}}/\mathrm{mL})\times {\rm{fasting}}\,{\rm{glucose}}\,(\mathrm{mmol}/{\rm{L}})]/22.5$$

The levels of TSH, FT4 and anti-thyroperoxidase antibody (anti-TPO Ab) were assessed with an electrochemiluminescence immunoassay (E-602; Roche, Germany). The normal reference ranges of TSH and FT4 were 0.4–4.0 mIU/L and 0.9–2.0 ng/mL, respectively.

### Lifestyle-related factors

Household income, smoking status, alcohol consumption, and physical activity were included in this study as lifestyle-related factors. Household income was recorded as quartiles from raw data from the KNHANES and classified into two groups (the lowest quartile or ≥ the second quartile). Smoking status was used to divide the patients into two groups (smoker or nonsmoker). The smoker group included current smokers or former smokers (participants who had smoked ≥100 cigarettes over their lifetime). Alcohol consumption was classified into two groups (drinker or nondrinker). A drinker was defined as a subject who drank ≥2 alcoholic beverages/month during the past year. Physical activity was used to divide subjects into two groups (yes or no), and subjects were included in the ‘yes’ group if they performed intense physical activity ≥20 min/day and ≥3 days/week, if they performed moderate physical activity ≥30 min/day and ≥5 days/week, or if they walked ≥30 min/day and ≥5 days/week.

### Definition of MetS and its components

MetS and the components of MetS were defined based on the National Cholesterol Education Program III (NCEP III) modified by Cook *et al*.^26^. The five mandatory components of MetS are an elevated WC, elevated BP, elevated glucose, elevated TG and reduced HDL-C. A WC ≥ the sex- and age-specific 90th percentile was the criterion for elevated WC. An SBP or DBP ≥ the sex-, age- and height-specific 90th percentile according to 2007 Korean reference charts^23^ or current treatment for hypertensive disorder were the criteria for elevated BP. A level of fasting glucose ≥110 mg/dL or a previously diagnosis of T2DM were the criteria for elevated glucose. Subjects with self-reported T2DM on a questionnaire (yes or no) or with current treatment using oral medications or subcutaneous insulin to manage T2DM were regarded as having T2DM. A serum TG level ≥110 mg/dL was the criterion for elevated TG, whereas a serum HDL-C level <40 mg/dL was the criterion for reduced HDL-C. Three or more of the five mandatory components constituted the presence of MetS.

### Statistical analyses

In the current study, R version 3.5.1 (The R Foundation for Statistical Computing, Vienna, Austria) was used for the statistical analyses. The clinical characteristics of the study population are presented according to sex. The means ± standard deviations (SDs) and percentages (%) are reported for the continuous variables and categorical variables. To determine significant differences, the independent t-test or Mann-Whitney U test and the chi-squared test were used for continuous variables and categorical variables, respectively. To analyze the correlations of TSH and FT4 with clinical parameters, Pearson’s correlation analyses were performed with no adjustment, and correlation coefficients (r) were calculated. The relationships of TSH and FT4 with cardiometabolic risk factors, including SBP, DBP, glucose, HbA1c, insulin, HOMA-IR, T-C, HDL-C, non-HDL-C, TG, the TG/HDL-C ratio, and LDL-C were assessed using partial correlation coefficients (r) after controlling for sex, age and BMI SDS. A multiple linear regression analysis between TSH and FT4 as independent variables and cardiometabolic risk factors was conducted after adjusting for possible confounders. In model 1, a multiple linear regression analysis of SBP and DBP was performed after adjustment for sex, age, BMI SDS, glucose, insulin, T-C, HDL-C, TG, LDL-C, anti-TPO Ab, and lifestyle-related factors including household income, smoking status, alcohol consumption, and physical activity. In model 2, a multiple linear regression analysis of glucose, insulin and HOMA-IR was performed after controlling for sex, age, BMI SDS, SBP, DBP, T-C, HDL-C, TG, LDL-C, anti-TPO Ab, and lifestyle-related factors. In model 3, a multiple linear regression analysis of T-C, HDL-C, non-HDL-C, TG, the TG/HDL-C ratio, and LDL-C was performed after adjusting for sex, age, BMI SDS, SBP, DBP, anti-TPO Ab, and lifestyle-related factors. The respective standardized regression coefficient (β) and standard error (SE) values were determined for each model. To evaluate the relationship between TSH and FT4 within reference ranges and MetS and its component, means of levels of TSH and FT4 were determined with no adjustment in model 4. In model 5, adjusted means of TSH and FT4 were assessed after adjustment for possible confounders such as sex, age, BMI SDS, anti-TPO Ab, and lifestyle-related factors using analysis of covariance (ANCOVA), which are presented as the means ± standard errors (SE). Statistical significance was considered when the *P* value was < 0.05.

## Results

### Clinical characteristics of the study population

The clinical characteristics of the subjects are shown in Table 1. One hundred thirty-one children and adolescents were males, and 119 children and adolescents were females. Males had a higher mean height SDS (0.75 vs 0.46, *P* = 0.039), SBP (111.35 mmHg vs 106.35 mmHg, *P* < 0.001), glucose (92.89 mg/dL vs 90.87 mg/dL, *P* = 0.019), and FT4 (1.34 ng/mL vs 1.27 ng/mL, *P* = 0.004) than females. Males were more likely to be smokers (*P* = 0.016).

**Table 1:** Clinical characteristics of the study population (*n* = 250).

	Males	Females	*P*
	(*n* = 131)	(*n* = 119)	
Age (years)	14.40 ± 2.54	14.13 ± 2.54	0.388
Height SDS	0.75 ± 1.07	0.46 ± 1.10	0.039
Weight SDS	0.54 ± 1.10	0.39 ± 1.21	0.299
WC SDS	0.12 ± 1.11	0.09 ± 1.09	0.855
BMI SDS	0.26 ± 1.11	0.24 ± 1.13	0.889
SBP (mmHg)	111.35 ± 9.03	106.35 ± 7.92	<0.001
DBP (mmHg)	65.90 ± 7.82	66.77 ± 7.46	0.373
Glucose (mg/dL)	92.89 ± 6.91	90.87 ± 6.50	0.019
HbA1c (%)	5.31 ± 0.26	5.31 ± 0.28	0.825
Insulin (μU/mL)	12.61 ± 7.08	13.70 ± 9.00	0.287
HOMA-IR	2.92 ± 1.68	3.13 ± 2.22	0.398
T-C (mg/dL)	158.42 ± 26.97	164.11 ± 24.07	0.081
HDL-C (mg/dL)	50.70 ± 10.46	52.10 ± 9.22	0.265
Non-HDL-C (mg/dL)	107.72 ± 26.12	112.01 ± 22.91	0.171
TG (mg/dL)	83.42 ± 57.72	87.75 ± 42.44	0.504
TG/HDL-C ratio	1.85 ± 1.69	1.80 ± 1.12	0.789
LDL-C (mg/dL)	91.07 ± 24.03	94.47 ± 22.63	0.251
TSH (mIU/L)	2.23 ± 0.81	2.11 ± 0.85	0.276
FT4 (ng/mL)	1.34 ± 0.17	1.27 ± 0.17	0.004
Anti-TPO Ab (U/mL)	8.83 ± 18.73	11.23 ± 29.55	0.441
Household income (≤lowest quartile, %)	12 (9.2%)	21 (17.6%)	0.176
Smoker (%)	23 (17.6%)	8 (6.7%)	0.016
Drinker (%)	6 (4.6%)	5 (4.2%)	0.999
Physical activity (%)	74 (56.5%)	67 (56.3%)	0.999
MetS and its components			
Elevated WC	18 (13.7%)	16 (13.5%)	>0.999
Elevated BP	22 (16.8%)	26 (21.9%)	0.394
Elevated glucose	1 (0.8%)	1 (0.8%)	>0.999
Elevated TG	28 (21.4%)	27 (22.7%)	0.922
Reduced HDL-C	18 (13.7%)	12 (10.1%)	0.488
MetS	9 (6.9%)	4 (3.4%)	0.336

Data are presented as the mean ± SD (standard deviation).

SDS, standard deviation score; WC, waist circumference; BMI, body mass index; SBP, systolic blood pressure; DBP, diastolic blood pressure; HbA1c, glycosylated hemoglobin; HOMA-IR, homeostatic model assessment for insulin resistance; T-C, total cholesterol; HDL-C, high-density lipoprotein cholesterol; TG, triglycerides; LDL-C, low-density lipoprotein cholesterol; TSH, thyroid-stimulating hormone; FT4, free thyroxine; anti-TPO Ab, anti-thyroperoxidase antibody; MetS, metabolic syndrome; BP, blood pressure.

### Unadjusted and adjusted correlations of TSH and FT4 with cardiometabolic risk factors

The unadjusted correlations of TSH and FT4 with clinical parameters are presented in Table 2. TSH levels were positively correlated with glucose (r = 0.172, *P* = 0.006), HbA1c (r = 0.149, *P* = 0.018), insulin (r = 0.144, *P* = 0.023), HOMA-IR (r = 0.163, *P* = 0.010), and TG (r = 0.155, *P* = 0.014). FT4 levels were significantly inversely correlated with sex (female, r = −0.183, *P* = 0.004), WC SDS (r = −0.133, *P* = 0.033), BMI SDS (r = −0.126, *P* = 0.046), insulin (r = −0.219, *P* < 0.001), and HOMA-IR (r = −0.122, *P* < 0.001), whereas FT4 concentrations were positively but not significantly correlated with TG (r = −0.211, *P* = 0.055). In addition, the adjusted correlations of TSH and FT4 with clinical parameters were calculated after controlling for sex, age, and BMI SDS. Table 3 shows the adjusted correlations of TSH and FT4 with cardiometabolic risk factors. TSH levels were positively correlated with glucose (r = 0.166, *P* = 0.009), HbA1c (r = 0.146, *P* = 0.022), insulin (r = 0.147, *P* = 0.021), HOMA-IR (r = 0.168, *P* = 0.008), and TG (r = 0.152, *P* = 0.017). FT4 levels were inversely correlated with insulin (r = −0.169, *P* = 0.008) and HOMA-IR (r = −0.163, *P* = 0.010).

**Table 2:** Unadjusted correlations of thyroid-stimulating hormone (TSH) and free thyroxine (FT4) with clinical parameters in euthyroid Korean children and adolescents aged 10–18 years (*n* = 250).

	TSH (μU/mL)		FT4 (ng/mL)	
	r	*P*	r	*P*
Sex (Female)	−0.069	0.276	−0.183	0.004
Age (years)	0.016	0.804	0.027	0.672
Height SDS	0.047	0.460	−0.017	0.792
Weight SDS	0.060	0.341	−0.101	0.111
WC SDS	0.067	0.293	−0.134	0.035
BMI SDS	0.054	0.398	−0.126	0.046
SBP (mmHg)	0.043	0.498	−0.017	0.793
DBP (mmHg)	−0.024	0.703	0.018	0.775
Glucose (mg/dL)	0.172	0.006	−0.030	0.642
HbA1c (%)	0.149	0.018	−0.035	0.582
Insulin (μU/mL)	0.144	0.023	−0.219	<0.001
HOMA-IR	0.163	0.010	−0.211	<0.001
T-C (mg/dL)	0.008	0.900	−0.101	0.113
HDL-C (mg/dL)	0.006	0.922	0.018	0.775
Non-HDL-C (mg/dL)	0.006	0.927	−0.112	0.077
TG (mg/dL)	0.155	0.014	−0.122	0.055
TG/HDL-C ratio	0.131	0.038	−0.100	0.114
LDL-C (mg/dL)	−0.061	0.340	−0.064	0.328
TSH (mIU/L)	—	—	−0.050	0.428
FT4 (ng/mL)	−0.050	0.428	—	—
Anti-TPO Ab (U/mL)	0.035	0.586	0.079	0.214

TSH, thyroid-stimulating hormone; FT4, free thyroxine; SDS, standard deviation score; WC, waist circumference; BMI, body mass index; SDS, standard deviation score; SBP, systolic blood pressure; DBP, diastolic blood pressure; HbA1c, glycosylated hemoglobin; HOMA-IR, homeostatic model assessment for insulin resistance; T-C, total cholesterol; HDL-C, high-density lipoprotein cholesterol; TG, triglycerides; LDL-C, low-density lipoprotein cholesterol; anti-TPO Ab, anti-thyroperoxidase antibody.

**Table 3:** Adjusted correlations of thyroid-stimulating hormone (TSH) and free thyroxine (FT4) with cardiometabolic risk factors after adjustment for sex, age, and body mass index (BMI) and standard deviation score (SDS) in euthyroid Korean children and adolescents aged 10–18 years (*n* = 250).

	TSH (μU/mL)		FT4 (ng/mL)	
	r	*P*	r	*P*
SBP (mmHg)^1^	0.004	0.951	−0.038	0.554
DBP (mmHg)^1^	−0.033	0.605	0.035	0.582
Glucose (mg/dL)^2^	0.166	0.009	−0.037	0.558
HbA1c (%)^2^	0.146	0.022	−0.021	0.747
Insulin (μU/mL)^2^	0.147	0.021	−0.169	0.008
HOMA-IR^2^	0.168	0.008	−0.163	0.010
T-C (mg/dL)^3^	0.009	0.886	−0.070	0.273
HDL-C (mg/dL)^3^	0.023	0.720	0.007	0.915
Non-HDL-C (mg/dL)^3^	0.001	0.994	−0.077	0.230
TG (mg/dL)^3^	0.152	0.017	−0.100	0.117
TG/HDL-C ratio^3^	0.124	0.053	−0.088	0.168
LDL-C (mg/dL)^3^	−0.065	0.306	−0.035	0.582

TSH, thyroid-stimulating hormone; FT4, free thyroxine; SBP, systolic blood pressure; DBP, diastolic blood pressure; HbA1c, glycosylated hemoglobin; HOMA-IR, homeostatic model assessment for insulin resistance; T-C, total cholesterol; HDL-C, high-density lipoprotein cholesterol; TG, triglycerides; LDL-C, low-density lipoprotein cholesterol; anti-TPO Ab, anti-thyroperoxidase antibody.

### Multiple linear regression analyses of TSH and FT4 with cardiometabolic risk factors

To evaluate the associations of TSH and FT4 with cardiometabolic risk factors, a multiple linear regression analysis was performed. The results of the multiple linear regression analyses are presented in Table 4. TSH was significantly positively associated with glucose (β = 1.179, *P* = 0.021), HbA1c (β = 0.044, *P* = 0.039) and TG (β = 8.158, *P* = 0.041), whereas TSH was positively but not significantly associated with HOMA-IR (β = 0.232, *P* = 0.060). FT4 was inversely associated with serum fasting insulin (β = −5.884, *P* = 0.017) and HOMA-IR (β = −1.364, *P* = 0.023).

**Table 4:** Multivariate linear regression analyses of thyroid-stimulating hormone (TSH) and free thyroxine (FT4) with cardiometabolic risk factors in euthyroid Korean children and adolescents aged 10–18 years (*n* = 250).

	TSH (mIU/L)			FT4 (ng/mL)		
	β	SE	*P*	β	SE	*P*
SBP (mmHg)^a^	−0.315	0.631	0.618	−0.731	3.052	0.811
DBP (mmHg)^a^	−0.289	0.563	0.609	2.293	2.721	0.400
Glucose (mg/dL)^b^	1.179	0.506	0.021	−1.082	2.454	0.660
HbA1c (%)^b^	0.044	0.021	0.039	−0.012	0.100	0.907
Insulin (μU/mL)^b^	0.779	0.505	0.124	−5.884	2.449	0.017
HOMA-IR^b^	0.232	0.123	0.060	−1.364	0.596	0.023
T-C (mg/dL)^c^	0.265	1.984	0.894	−10.400	9.677	0.284
HDL-C (mg/dL)^c^	0.317	0.773	0.682	0.587	3.771	0.876
Non-HDL-C (mg/dL)^c^	−0.052	1.881	0.978	−10.987	9.172	0.232
TG (mg/dL)^c^	8.158	3.969	0.041	−29.740	19.352	0.126
TG/HDL-C ratio^c^	0.193	0.113	0.091	−0.730	0.553	0.188
LDL-C (mg/dL)^c^	−1.660	1.799	0.357	−4.879	8.773	0.579

TSH, thyroid-stimulating hormone; FT4, free thyroxine; β, standardized regression coefficient; SE, standard error SBP, systolic blood pressure; DBP, diastolic blood pressure; HOMA-IR, homeostatic model assessment for insulin resistance; T-C, total cholesterol; HDL-C, high-density lipoprotein cholesterol; TG, triglycerides; LDL-C, low-density lipoprotein cholesterol.

^a^A multivariate linear regression analysis for the associations of systolic blood pressure (SBP) and diastolic blood pressure (DBP) as dependent variables and thyroid-stimulating hormone (TSH) and free thyroxine (FT4) as independent variables was performed after adjustment for possible confounders, including sex, age, body mass index (BMI) standard deviation scores (SDSs), glucose, insulin, total cholesterol (T-C), high-density lipoprotein cholesterol (HDL-C), triglycerides (TG), low-density lipoprotein cholesterol (LDL-C), anti-thyroperoxidase antibody (anti-TPO Ab), household income, smoking status, alcohol consumption, and physical activity.

^b^A multivariate linear regression analysis for the associations of serum fasting glucose, serum fasting insulin and the homeostatic model assessment of insulin resistance (HOMA-IR) as dependent variables and TSH and FT4 as independent variables was performed after adjustment for possible confounders including sex, age, BMI SDS, SBP, DBP, T-C, HDL-C, TG, LDL-C, anti-TPO Ab, household income, smoking status, alcohol consumption, and physical activity.

^c^A multivariate linear regression analysis for the association of T-C, HDL-C, TG, and LDL-C as dependent variables and TSH and FT4 as independent variables was performed after adjustment for possible confounders, including sex, age, BMI SDS, SBP, DBP, anti-TPO Ab, household income, smoking status, alcohol consumption, and physical activity.

### Unadjusted and adjusted mean TSH and FT4 according to the presence of MetS and its component

The unadjusted and adjusted mean TSH and FT4 are presented in Table 5. In unadjusted model 4, children and adolescents with elevated glucose had a higher mean TSH compared to those without elevated glucose (3.87 mIU/L vs 2.16 mIU/L, *P* = 0.004). In adjusted model 5, participants with elevated glucose had an elevated mean TSH compared to those without elevated glucose (3.88 mIU/L vs 2.16 mIU/L, *P* = 0.005) after controlling for possible confounders using ANCOVA.

**Table 5:** The unadjusted and adjusted means of thyroid-stimulating hormone (TSH) and free thyroxine (FT4) according to the presence of metabolic syndrome (MetS) and its components in euthyroid Korean children and adolescents aged 10–18 years (*n* = 250).

	No elevated WC^4^	Elevated WC^4^	*P*	No elevated WC^5^	Elevated WC^5^	*P*
	(*n* = 216)	(*n* = 34)		(*n* = 216)	(*n* = 34)	
TSH (mIU/L)	2.16 ± 0.84	2.25 ± 0.81	0.572	2.17 ± 0.06	2.17 ± 0.17	0.995
FT4 (ng/mL)	1.31 ± 0.17	1.26 ± 0.16	0.096	1.31 ± 0.01	1.28 ± 0.04	0.514
	**No elevated BP** ^**4**^	**Elevated BP** ^**4**^	***P***	**No elevated BP** ^**5**^	**Elevated BP** ^**5**^	***P***
	**(*n*** = **202)**	**(*n*** = **48)**		**(*n*** = **202)**	**(*n*** = **48)**	
TSH (mIU/L)	2.16 ± 0.84	2.21 ± 0.82	0.714	2.16 ± 0.06	2.21 ± 0.12	0.693
FT4 (ng/mL)	1.30 ± 0.18	1.31 ± 0.15	0.804	1.30 ± 0.01	1.32 ± 0.03	0.442
	**No elevated glucose** ^**4**^	**Elevated glucose** ^**4**^	***P*** ^**a**^	**No elevated glucose** ^**5**^	**Elevated glucose** ^**5**^	***P***
	**(*n*** = **248)**	**(*n*** = **2)**		**(*n*** = **248)**	**(*n*** = **2)**	
TSH (mIU/L)	2.16 ± 0.82	3.87 ± 0.06	0.004	2.16 ± 0.05	3.88 ± 0.59	0.004
FT4 (ng/mL)	1.31 ± 0.17	1.27 ± 0.09	0.804	1.31 ± 0.01	1.33 ± 0.12	0.860
	**No elevated TG** ^**4**^	**Elevated TG** ^**4**^	***P***	**No elevated TG** ^**5**^	**Elevated TG** ^**5**^	***P***
	**(*n*** = **195)**	**(*n*** = **55)**		**(*n*** = **195)**	**(*n*** = **55)**	
TSH (mIU/L)	2.12 ± 0.83	2.35 ± 0.84	0.070	2.13 ± 0.06	2.31 ± 0.11	0.185
FT4 (ng/mL)	1.32 ± 0.18	1.27 ± 0.15	0.090	1.31 ± 0.01	1.27 ± 0.02	0.119
	**No reduced HDL-C** ^**4**^	**Reduced HDL-C** ^**4**^	***P***	**No reduced HDL-C** ^**5**^	**Reduced HDL-C** ^**5**^	***P***
	**(*n*** = **220)**	**(*n*** = **30)**		**(*n*** = **220)**	**(*n*** = **30)**	
TSH (mIU/L)	2.17 ± 0.82	2.20 ± 0.91	0.865	2.17 ± 0.06	2.19 ± 0.15	0.921
FT4 (ng/mL)	1.31 ± 0.17	1.30 ± 0.17	0.851	1.30 ± 0.01	1.31 ± 0.03	0.865
**MetS**	**No MetS** ^**4**^	**MetS** ^**4**^	***P*** ^**a**^	**No MetS** ^**5**^	**MetS** ^**5**^	***P***
	**(*n*** = **237)**	**(*n*** = **13)**		**(*n*** = **237)**	**(*n*** = **13)**	
TSH (mIU/L)	2.15 ± 0.83	2.59 ± 0.80	0.060	2.15 ± 0.05	2.54 ± 0.24	0.124
FT4 (ng/mL)	1.31 ± 0.18	1.22 ± 0.09	0.003	1.31 ± 0.01	1.23 ± 0.05	0.113

Data are presented as the means ± standard deviation (SD) in model 4 and means ± standard error (SE) in model 5.

WC, waist circumference; TSH, thyroid-stimulating hormone; FT4, free thyroxine; BP, blood pressure; TG, triglyceride; HDL-C, high-density lipoprotein cholesterol; MetS, metabolic syndrome.

^4^Means ± SD of MetS and its component were determined with no adjustment.

^5^Means ± SE of MetS and its component were assessed after adjustment for sex, age, BMI SDS, anti-TPO Ab, household income, smoking status, alcohol consumption, and physical activity.

^a^Statistical significance was determined using the Mann-Whitney U test.

## Discussion

In our nationally representative population-based study, high TSH and/or low FT4 values within the reference ranges, which are indicative of low-normal thyroid function, were significantly related to increased cardiometabolic risk factors. TSH was significantly positively correlated with glucose, HbA1c, insulin, HOMA-IR, and TG, whereas FT4 was significantly inversely correlated with WC SDS, BMI SDS, insulin and HOMA-IR in unadjusted correlation analyses. These relationships remained statistically significant after adjusting for sex, age, and BMI SDS. In addition, TSH was significantly positively associated with glucose, HbA1c, and TG, whereas FT4 was significantly inversely related to insulin and HOMA-IR in the fully adjusted model using multiple linear regression analyses after controlling for possible confounders. In covariance analyses, boys and girls with elevated glucose had a higher level of TSH within reference ranges compared to those without elevated glucose after adjustment for possible confounders.

With regard to lipid profiles, the results of previous studies regarding the relationship between TSH and lipid status in euthyroid children and adolescents had some discrepancies. A U.S. study reported a significantly positive link between TSH and T-C and a positive but nonsignificant trend between TSH and TG in euthyroid adolescents aged 12–18 years^27^. A U.S. study in euthyroid children and adolescents aged 2–18 years reported a positive association of TSH with TG and an inverse correlation between FT4 and TG^28^. In addition, a German study demonstrated that TSH levels within the reference range are positively associated with T-C, LDL-C, and TG^29^. The present study revealed a positive relationship between TSH and TG, although the link between FT4 and lipid profile results was not significant. The relationship between TSH and TG remained significant after adjustment for possible confounders.

With regard to the relationships among thyroid hormones, glucose metabolism and insulin resistance, FT4 was significantly inversely correlated with insulin and HOMA-IR. However, TSH was significantly positively correlated with glucose and HbA1c in multiple linear regression analyses after controlling for confounders. A study in 36 euthyroid obese adolescents aged 12–18 years demonstrated that there was a sex-specific association between TSH and insulin sensitivity and that TSH was significantly inversely related to the insulin sensitivity index in boys but not in girls^30^. A U.S. report demonstrated that TSH, FT3, and the FT3-to-FT4 ratio are significantly correlated with fasting glucose and HOMA-IR, whereas FT3 and the FT3-to-FT4 ratio are related to fasting insulin (no relationship between TSH and fasting insulin)^27^. In a Mexican prospective study involving 100 euthyroid children at high risk for T2DM, FT4 was significantly inversely related to fasting insulin and HOMA-IR, whereas TSH was not correlated with fasting glucose, fasting insulin or HOMA-IR^31^. Moreover, a study involving children and adolescents undergoing a two-month intensive weight reduction program showed that decreases in TSH levels compared to baseline concentrations could predict a reduction in fasting insulin and HOMA-IR after adjustment for age, sex, and body composition, although changes in FT4 could not^32^.

With regard to MetS and its components, a few studies regarding the relationship of low-normal thyroid function within the reference range with MetS and its components were conducted in the adult population. A Dutch study demonstrated negative relationships between FT4 levels within the reference range and elevated WC, elevated glucose, elevated TG, and reduced HDL-C in adults^33^. In their study, a positive association of TSH with TG was observed after adjustment for sex and age^33^. A U.S. study showed a relationship between higher TSH within the reference range and an increase in the prevalence of MetS in healthy adults^34^. A Korean study revealed that lower FT4 levels within the reference range were related to the presence of MetS in men and women, but this relationship was not significant after adjustment for age^35^. Some studies in children and adolescents were conducted to evaluated the link between thyroid function and MetS. In a recent study, Japanese boys with MetS tended to have a higher mean FT4-to-FT3 ratio but the difference was not statistically significant (*P* = 0.05)^36^. In a Turkish study, children with MetS exhibited elevated TSH levels compared with those without MetS^37^. However, neither the Japanese nor Turkish studies were conducted in a study population with normal thyroid function, and children and adolescents with SCH (TSH ≥4.0 mIU/L) were included in their studies^36,37^. This study demonstrated that a higher level of TSH within the reference range was related to the presence of elevated glucose after adjustment for confounders in euthyroid boys and girls.

The current study has a few limitations. First, our study was a cross-sectional study, and causality between low-normal thyroid function test results and cardiometabolic risk factors could not be proven. Second, we could not obtain information regarding the familial history of thyroid disease and cardiometabolic diseases, including T2DM, hypertension, dyslipidemia and MetS. Third, we could not analyze data regarding pubertal development (Tanner stage), which may play a role as a confounding factor. Thyroid hormone levels may be influenced by the progression of puberty, although the relationship between pubertal development and thyroid hormone may be attenuated in boys compared to girls^38,39^. It is also well established that insulin resistance can be influenced by puberty^40^. However, age may provide a rough approximation of pubertal progression. In the present analysis, FT4 was significantly inversely related to insulin resistance, and TSH was significantly positively correlated with fasting glucose levels and HbA1c after adjustment for possible confounders. Finally, we could not show relationships between TSH, FT4 and MetS and its components excluding elevated glucose. This difficulty may be related to the small study population affected by MetS and its components. Nevertheless, we could present a significant correlation of TSH and FT4 with cardiometabolic risk factors. Future large-scale studies should be performed to demonstrate relationships between low-normal thyroid function within the reference ranges and MetS and its components in children and adolescents.

In conclusion, the current Korean nationally representative population-based study showed that high TSH within the reference range was significantly associated with increases in serum fasting glucose, HbA1c and TG, whereas low FT4 within the reference range was related to increases in serum fasting insulin and an insulin resistance index (HOMA-IR) even after controlling for possible confounders in euthyroid children and adolescents aged 10–18 years. In addition, males and females aged 10–18 years with elevated glucose exhibited a higher TSH level within reference ranges compared to those without elevated glucose after adjustment for confounding factors using ANCOVA. Our results suggest that low-normal thyroid function test results, even within the reference ranges, may be related to increased cardiometabolic risk factors. Future longitudinal prospective studies are indicated to evaluate whether low-normal thyroid function test results may be indicators of early identification for individuals at high risk for cardiometabolic disease.
